# *“I loved it, absolutely loved it”* a qualitative study exploring what student podiatrists learn volunteering as part of an interprofessional medical team at a marathon

**DOI:** 10.1186/s13047-023-00607-1

**Published:** 2023-02-20

**Authors:** Simon Otter, Deborah Whitham, Paula Riley, James Coughtrey, Sophia Whitham

**Affiliations:** 1 Centre for Regenerative Medicine & Devices, School of Applied Sciences, University of Brighton, Huxley Building Lewes Road, Brighton, BN2 4GJ UK; 2 AECC University College, Parkwood Campus, Parkwood Road, Bournemouth, Dorset, BH5 2DF UK; 3 School of Sport & Health Sciences, University of Brighton, 49 Darley Rd, Eastbourne, BN20 7UR UK; 4 Royal College of Podiatry, Quartz House, 207 Providence Square, Mill Street, London, SE1 2EW UK; 5 Gills Farm, London Rd, Battle, TN33 0LS UK

**Keywords:** Qualitative research, Volunteering, Interprofessional, Podiatry, Marathon

## Abstract

**Background:**

Final year podiatry students volunteer annually as part of the wider interprofessional medical team at both the Brighton and London Marathon race events, supervised by qualified podiatrists, allied health professionals and physicians. Volunteering has been reported to be a positive experience for all participants and a way of developing a range of professional, transferable, and where appropriate, clinical skills. We sought to explore the lived experience of 25 students who volunteered at one of these events and aimed to: i) examine the experiential learning reported by students while volunteering in a dynamic and demanding clinical field environment; ii) determine whether there were elements of learning that could be translated to the traditional teaching environment in a pre-registration podiatry course.

**Methods:**

A qualitative design framework informed by the principles of interpretative phenomenological analysis, was adopted to explore this topic. We used IPA principles to enable analysis of four focus groups over a two-year period to generate findings. Focus group conversations were led by an external researcher, recorded, independently transcribed verbatim and anonymised prior to analysis by two different researchers. To enhance credibility, data analysis was followed by independent verification of themes, in addition to respondent validation.

**Results:**

In total, five themes were identified: i) a new inter-professional working environment, ii) identification of unexpected psychosocial challenges, iii) the rigors of a non-clinical environment, iv) clinical skill development, and v) learning in an interprofessional team.

**Summary:**

Throughout the focus group conversations, a range of positive and negative experiences were reported by the students. This volunteering opportunity fills a gap in learning as perceived by students, particularly around developing clinical skills and interprofessional working. However, the sometimes-frantic nature of a Marathon race event can both facilitate and impede learning. To maximize learning opportunities, particularly in the interprofessional environment, preparing students for new or different clinical settings remains a considerable challenge.

**Supplementary Information:**

The online version contains supplementary material available at 10.1186/s13047-023-00607-1.

## Introduction

Podiatrists focus on the foot and lower limb seeking to enhance mobility and independence and to improve quality of life for their patients [1]. In the UK, professional registration can be granted following a degree programme offered by a recognised provider [2]. Historically, podiatric clinical education has been somewhat uni-professional, concentrated in tertiary educational institutions offering the specialism [3]. Student podiatrists are educated in the theoretical basis on which to ground treatment decisions and are also required to develop highly specialised and detailed psychomotor skills to meet professional benchmarks [4]. Clinical learning is typically situated within university-hosted clinics, enabling students to gain experience and confidence, particularly in ‘soft skills’, prior to external placements in workplace settings [5, 6]. The increased complexity of contemporary healthcare means that interprofessional teams are common in the workplace and exposure to cross-professional working is advocated during undergraduate podiatry education [7]. To ensure work-readiness, interprofessional education seeks to facilitate graduates who are embedded in collaborative practice, yet interprofessional placements are under-reported in the literature [8]. In clinical practice, interprofessional teamwork is seen as essential to achieving the shared goal of improving the delivery of quality healthcare services, particularly for those people with complex, long-term conditions [9, 10], conditions that are commonly seen in podiatric practice. Consequently, new models around interprofessional placements are an increasingly important requirement [1, 11].

A recent systematic review highlighted that healthcare professionals seek to enhance collaboration by bridging role-specific gaps, collaborating on overlaps in roles and enabling the space and capacity to undertake these changes [12]. As outlined above, the historic uni-professional approach to podiatric clinic education is being replaced by more diverse placements in both NHS and third sector organisations seek to better prepare students for life post-qualification. One example of the type of activity that seeks to embed interdisciplinary working is seen during mass participation mega sports events, such as marathons, which are typically staffed by volunteer medical professionals and charitable organisations [13, 14], who must work together to manage a dynamic and complex environment [15]. Marathon running is associated with a wide range of musculoskeletal injuries including medial tibia stress syndrome, Achilles tendinopathy and plantar fasciitis [16, 17]. These conditions are often managed by podiatrists. However, when presenting in the context of the arduous physical demands post- marathon, often a more interprofessional approach is required as several complaints co-exist, for example an acute musculoskeletal injury, foot blisters and muscle cramps. Clinicians need to rapidly identify each pathology and determine an efficient management plan. The limited resources for multiple patients with a wide range of diverse needs, together with minimal patient background information, means clinicians must work together, adapting and delivering treatments using different parameters to their usual clinical routine/environment, where clinicians have access to records and relevant additional diagnostic information [18].

Podiatry students have been volunteering at UK marathon events for over 20 years and work in a supervised interprofessional environment alongside qualified professionals from different disciplines (e.g., podiatrists, physiotherapists and physicians) to provide effective immediate care for a range of lower limb injuries. While examples of interprofessional learning in podiatry exist [19], these are under-represented in the literature. There is also a paucity of reporting on volunteering as a learning opportunity within the allied health professions in general. Reports often limited to newsworthy items or case histories in professional journals as opposed to formal empirical investigations. Yet, volunteering is acknowledged as an excellent way to gain experience in a wide range of settings, with opportunities for participants to improve skills in an interprofessional setting, develop self-esteem, enhance wellbeing and benefit from the social engagement [20]. Volunteering is often seen as a ‘win-win’ situation, with benefits for those who volunteer in terms of both skill acquisition and the development of social and professional networks [21, 22]. There are often opportunities for volunteers to develop interpersonal proficiencies to complement professional skills [23, 24]. There is a perceived intrinsic value/advantage to volunteering in terms of skill/knowledge acquisition – particularly of transferable skills developed while working in different settings [21–24]. Recipients of treatments provided by volunteers may often benefit from knowledge, skills and care that complement, and may exceed, what might ordinarily be expected [25]. The key advantage of volunteering in different settings is that it offers learners an exposure to new authentic environments that support interprofessional clinical learning. Environments that are otherwise impossible to replicate in traditional education settings [24]. However, the inherent advantages of volunteering in terms of interprofessional learning and transferrable skill development, risk being tempered by the increasing significance rightly placed on meeting student expectations regarding what they will learn in any given placement and how these expectations map to professional benchmarks [26, 27]. We aimed to examine the lived learning experience of podiatry students working in a volunteer capacity at an interprofessional clinical setting, during the Brighton and London marathon race events. Our specific objectives were: i) to examine the experiential learning reported by students while volunteering in a dynamic and demanding clinical field environment; ii) to determine whether there were elements of learning that could be translated to the traditional teaching environment in a pre-registration podiatry course.

## Methodology

### Study design

We designed a qualitative study informed by the principles of interpretative phenomenological analysis (IPA), [28, 29] seeking to highlight lived experience from individuals from a homogenous sample with a shared perspective [30, 31]. We employed a hermeneutic phenomenological approach to generate in-depth and illustrative information, to uncover what our participants gained from their personal volunteering experience and how their learning in this particular context might be translated into the wider clinical teaching environment [32–34].

### Subjects & settings

Participants in this study comprised two cohorts of podiatry students in their final year who had volunteered to participate as part of the wider interprofessional medical team at either the Brighton or London marathon events during 2018 and 2019 (Covid-19 restrictions preventing further data generation in 2020 and early 2021). To limit confounding variables associated with different medical stations and/or due to differences in academic preparation from different universities, this work was focused on students from a single institution who were based at the same medical station at one marathon event (i.e. Brighton or London). We collected data over a two-year period to limit any potential bias from a particular cohort effect. Recruitment took place via purposive sampling using posters in university buildings and student cohort emails to alert potential participants to the study and invite them to take part. Care was taken to send emails from administrative staff and not academic tutors to avoid coercion. Data were generated from four focus groups (total *n* = 25 participants), each taking place shortly after students had completed their volunteering experience.

### Ethical considerations & reflexivity

Ethical approval was granted by the University of Brighton, School of Health Sciences Research Governance and Ethics Panel. At the start of this project, three members of the research team (SO, DW, PR) were members of staff teaching in the institution where the research took place. We recognise our roles may have influenced our analysis of the data and the recommendations we made. We took steps to utilise researchers independent of the university to generate data and thereby, mitigate potential bias. Throughout, our approach we sought to ensure strict confidentiality and anonymity were maintained.

### Activity description

**Fig. 1:**
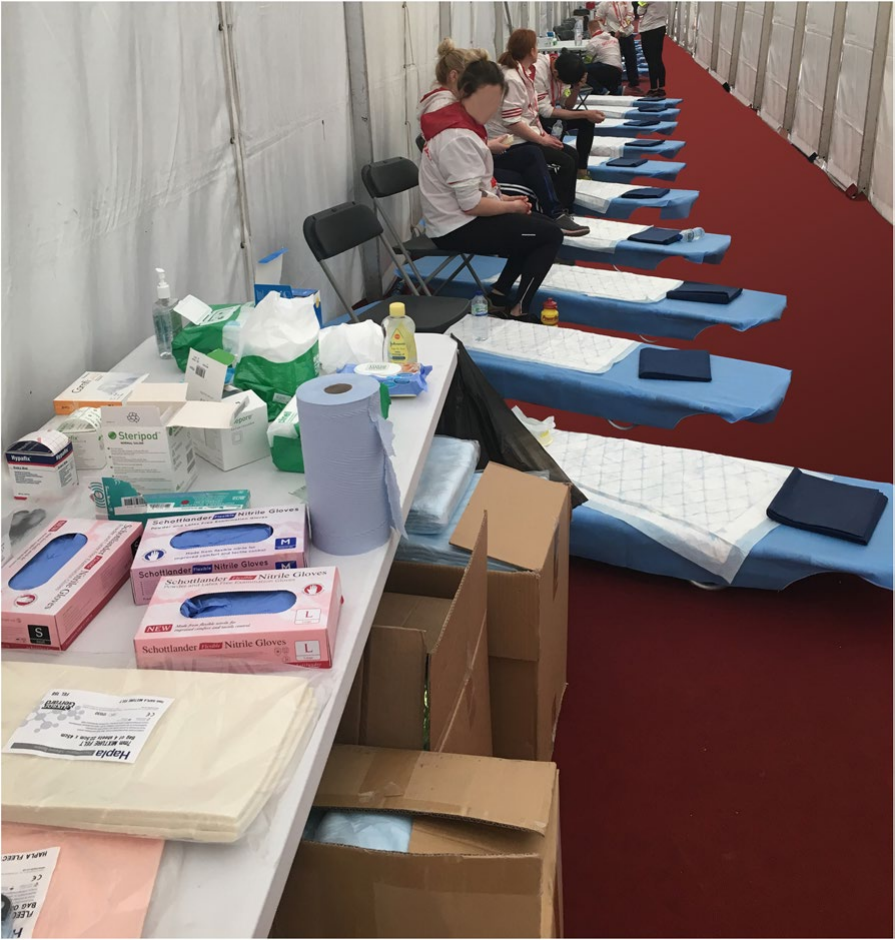
Clinical environment at the London Marathon. This image depicts one of the treatment tents at the London marathon prior to runners being admitted

Mass participation sporting events have a large (and typically unseen) group of health professionals (e.g., nurses, physicians, physiotherapists, podiatrists and voluntary agencies) who provide medical care for athletes taking part, based on well-established guidelines [35–37]. Student volunteers receive face-to-face and email briefing at their home University, prior to the event. At the event itself there are briefings for the medical team as a whole and further briefings at each medical station. These stations (Fig. 1) are tents with a series of stretchers and chairs for participants to sit/lie while care is delivered. In common with other major mass participation events, the aims of care are to prevent a competitor’s complaints from worsening, facilitate onward progression to collect belongings, and meet family/friends [37–39]. Additionally there are mechanisms to facilitate removal to hospital for those with more serious injuries and/or for those whose overall condition deteriorates.

### Data generation

At the time of this study there was no previous empirical work to guide our data generation. Development of the focus group schedule utilised a questioning route [40] where draft questions were initially derived from free text comments extracted from evaluation sheets, completed by student volunteers at previous marathon events. Construct validity of the draft schedule was enhanced by inviting alumni who had attended previous marathons to review and comment on the applicability and suitability of questions. The final focus group schedule (Additional file 1) consisted of four broad areas that sought: i) to explore what participants felt they had gained, ii) how they learned, iii) what knowledge/skills were transferrable to other clinical settings and iv) how the event compared with their expectations. IPA is typically recommended when capturing in-depth, individual, personal experiences [30]. However, IPA has also been used with focus groups to explore specific instances and allow for a targeted focus [41]. In this instance we were particularly interested in a methodology that would enhance personal accounts, capitalise on peer-to-peer interactions and the rapport generated between students given their shared experiences.

### Focus group management

A total of four focus groups were convened, each with between 6-8 respondents. We sought relatively small focus group numbers to ensure each respondent had ample opportunity to contribute, adding to the richness of data [42]. Each group was facilitated by a researcher who was not a member of university staff (JC). Importantly, they were an experienced group moderator who also understood the dynamics of the London marathon. Informed, written consent was gained prior to participation, respondents were provided with an information sheet and completed a consent form returned directly to administrative staff. Focus groups were conducted on university grounds and scheduled to accommodate the students’ academic timetable. Each focus group was digitally recorded and transcribed verbatim. Participant names were recorded on paper by the facilitator in the chronological order in which they first spoke. The raw audio data files were transcribed verbatim, and participants allocated a pseudonym by a research assistant who was independent of the university staff team (SW). Transcribed comments for each respondent were also allocated a different colour for ease of differentiation during data analysis.

### Data analysis

To reduce potential bias, data analysis was performed independently by two members of the research team (SO & DW). Initially, each researcher completed a generic thematic analysis approach as recommended by Bruan & Clarke [43]. All transcripts for each focus group were read several times prior to data extraction to ensure the researchers achieved familiarity with the data. To maintain confidentiality, audio files were not listened to as this may have identified participants. To search for meanings and themes, each piece of extracted data was initially coded to ensure researchers could relocate its origin (for example focus group A, theme 1 and the event (LM=London Marathon)). In addition, which participant was speaking, and the line number(s) of text were included for ease of comparison. Data could be extracted as key words (with the context put in brackets where needed) or as whole phrases of text to demonstrate potential themes. This detailed data coding was performed to increase the robustness of the research process, allowing a clear and transparent audit trail back to the transcript for subsequent verification. Subsequently an IPA approach was incorporated to enable a dual analytic focus – both the thematic orientation, (the themes across all participants) and an idiographic approach to focus on unique details for an individual [34]. Within this latter stage we sought to highlight the relevance of a participant’s volunteer experience to clinical practice and placement education, as well as to identify the resonance of this experience for participants [44].

Once this process was complete, the researchers (DW, SO) discussed their coding and organised themes into a meaningful whole. Common themes relating to the study aims were developed across the four focus groups consistent with the process of constant comparison analysis [40]. Themes were derived to focus on the lived experience of students, with an openness to capturing the notion of volunteering within the unique environment in which participants were situated [45]. Owing to variability of external factors (e.g. impact of weather conditions) data saturation was not an aim of this study. Dependability of data analysis was addressed by verification of themes by a researcher who had not been involved in the data extraction process who reviewed findings (PR). To enhance overall credibility, themes were returned to participants for checking and no recommendations for change were received. This study is reported in accordance with the consolidated criteria for reporting qualitative research (COREQ) - a 32-item checklist to assist authors to report the key aspects of qualitative research associated with interviews or focus groups [46].

## Results

In total, five themes were identified: i) a new inter-professional working environment, ii) identification of unexpected psychosocial challenges, iii) rigors of a non-clinical environment, iv) clinical skill development, and v) learning in an interprofessional team. Exemplars from the transcripts were chosen to illustrate themes, based on their frequency and specificity. This provides a rich, textual description to accurately reflect the experience being described [40, 41]. To ensure confidentiality throughout, students’ demographic details are not reported to prevent inadvertent recognition. We have provided codes for each quotation listed, and these are detailed as follows: Focus group (G1, G2 etc.), line number (e.g. L142-5), student number (e.g. S4) and the event attended (e.g. LM = London marathon, BM = Brighton marathon).

### A new inter-professional working environment

For our student participants, this was the first time they had worked within a true interdisciplinary team. They welcomed the opportunity to play their part and felt privileged to be part of a prestigious event.

*“Like, I’m just a student on podiatry course and we’re helping out on one of the biggest events in London every year I felt very privileged”*

G1 L425 S2 LM

Students saw, first-hand, how collaboration between clinicians enabled better outcomes for athletes who typically presented with more than one complaint.

*“I loved it so much, I absolutely loved it, I thought it was amazing! Everything about it, the atmosphere, working with the doctors. Yes, a very, very positive experience and I’d love to do it again. In fact, I’ve signed up to run next year already!”*

G3 L129-135 S4 LM

*“It was really the first time we’d worked as part of a multidisciplinary team. I know on placement we sort of do, but we didn’t work with doctors and nurses, whereas this experience did include that, even first aiders and St. Johns… it was a really broad spectrum of healthcare professionals”*

G1 L47-53 S3 LM

Students were able to contribute positively to patient care and the treatment process, and in so doing, bridge gaps in their knowledge and experience. They seemed genuinely surprised that different professionals could work well together, even though they had never met. This shed a different light on interprofessional working. Prior to the event students appeared to expect some form of hierarchy between clinicians but throughout the marathon, but a helpful and supportive atmosphere was clearly noted. This valuable and productive environment would seem more in-keeping with the professional workplace that students were now able to anticipate post-qualification.

*“I felt there was a very good sense of camaraderie with everyone helping each other in the tent, especially the day before when we had the brief all the doctors were very supportive, and I didn’t feel there was any sense of hierarchy”*

L3 L187-181 S6 BM

Interestingly, those taking part in this study share their university campus with a range of other healthcare students and interprofessional education is promoted throughout their courses. Nevertheless, the inter-professional working element of this volunteering experience was seen as being different and better. This may be because of the supportive atmosphere and lack of barriers between professionals and the focus of a shared aim to facilitate rapid care and discharge home wherever possible. It is possible therefore, that patient-facing placements offer additional opportunities for shared learning than has previously been recognised and such opportunities are not being fully capitalised.

“*The fact that nursing and physio are all on this campus and we don’t really see each other at all. I think it is a bit… it could be improved… it’s a wasted opportunity… working with them in the capacity we have been trained for… more hands on with the patients together… its how healthcare practitioners work…”*

G1 L482-491 S4,S5 LM

### Identification of unexpected psychosocial challenges

Students in this study (including those with prior healthcare experience) were shocked by both the volume of runners requiring hands-on treatment and the wide range of presenting clinical complaints, not all of which were obvious physical injuries.

*“Pretty shocking. I myself wasn’t too shocked about it as I have previous experience in healthcare, so I have seen those sorts of things before, but I’m not sure about everyone else. It was very, very shocking to say the least”*

G3 L35-38 S1 LM

Considerable surprise was expressed at the emotional toll the event took on those runners taking part, over and above the expected physical exertion of running a marathon. Many runners take part to raise funds for organisations they have a deep and meaningful personal connection with

*“A lot of people who were confused… very worked up… crying and not knowing what was going on*”

G3 L72-76 S3 LM

One respondent simply reported how runners were:

“*exhausted, disorientated, bewildered”*

G1 L81 S5 LM

Consequently, all our students reported offering psychosocial support to competitors. For example, congratulating their effort, confirmation they were not a burden, and enabling acceptance that seeking care was not unexpected. Students reported drawing on the health psychology component of their education was a key component in their treatment of runners, as a holistic approach to managing presenting physical complaint(s). This approach appeared not to have been drawn on in as much depth during their clinical education so far.

### Rigors of a non-clinical environment

All found the clinical environment (Fig. 1) a significant challenge as they had no similar experience to draw on. Students agreed that maintaining good infection control, safe disposal of clinical waste (including contaminated sharps), patient positioning for safety and comfort, effective time management and maintaining safe working practices, all required considerable additional thought. Indeed, some commented that the environment coupled with the psychosocial challenges described above were so different to their usual clinical setting, that adequate preparation would be difficult.

*“It’s just one of those things that you learn just by being there, so if you got told beforehand I still don’t think it would have helped*”

G3 L256 S6 BM

*“you just cannot prepare for this experience”*

G3 L213 S6 BM

The environment took other, more unexpected, tolls. Many students found they were applying their knowledge of safe manual handing simply to enable runners to enter and exit the medical facilities unharmed, (i.e., without falling) which had a notable personal physical impact on student volunteers.

*“I mean we are not unfit, but I think all of us were suffering physically for two days afterwards - you felt like you had run the marathon the next day.”*

*G3 L280-281 S4 LM*

### Clinical skill development

Throughout students reported that they had been involved in the management of a diverse range of complaints. By way of example, Table 1 illustrates the range and number of injuries recalled by students from one marathon medical station. Students reported that they were able apply and hone their specialist clinical skills with a group of patients (runners) who they had seen far less frequently in their clinical education to date. This, together with the impact of time pressure reported below, enhanced their confidence in their own ability

*“The experience for me proved to be very, very different from a normal clinic. It was all about using your initiative. … As a student I still feel we are second guessing ourselves about how to approach treatment. On the day it was a very good experience but frantic at the same time. It was definitely something that I can look back on and say ‘look I learnt a lot from that”*

*G3 L115-120 S1 LM*

*“definitely more confident, like with blisters and when to lance them, we saw so much at* XXXX *and we never saw any in clinic, so I was a bit nervous about doing them* [blisters]…. *but after that day I feel more confident about when to lance or not, and what advice to give”*

G2 L256-260 S1 BM

**Table 1:** Conditions managed at one marathon

Category of presenting complaint	Numbers presenting
Blisters	39
Nail lesions	3
Cramp	65
Soft tissue injury (strain, tendonosis)	8
Bone/Joint injury (sprain, suspected fracture)	10
Wound care	3
Exhaustion	51
Collapse	25

This table outlines the complaints podiatry students recalled managing at one medical station at one marathon

Moreover, the wide range of transferable skills students were able to implement were reported to be of particular value. This was not only in terms of hands-on foot care such as padding, strapping and wound care, but additionally application of their understanding of physiology and general medical pathology (for example for those runners with constitutional needs around dehydration).

*“the applying of general* [medical] *knowledge was quite useful, you didn’t realise you had all this other knowledge and when you’re out doing different things; it’s not just 'podiatry'”*

G4 L315-317 S3 LM

Gaining confidence in skill acquisition was important to students. Many reported being shown new techniques by other clinicians that they were then able to apply successfully under close supervision. Gaining positive feedback from patients and other health professionals further boosted their confidence.

*“I didn’t feel like we were just assisting the physios, we just worked together really. I learnt a lot off them, especially massaging technique”*

G2 L28-30 S1 BM

*“I’d definitely do it again. I learnt a lot from it”*

G2 L81082 S4 LM

### Learning in an interprofessional team

For many of the student volunteers, the nature of the event coupled with the challenging environment, offered a diverse learning opportunity where a different approach to learning was reported. Students were providing care for a group of people who were new to them, both in terms of being an athlete with an acute injury and having limited background referral detail. It’s relevant to note that the students’ experience to date often focussed more on chronic pathology. Students reported learning from each other and from other medical team members. They found information from runners themselves invaluable to make up for the lack of information they were used to having available in a traditional clinical placement. Runners had often encountered their particular complaint/injury previously and were able to offer ideas and guidance. Counter-intuitively, the time-pressured nature of the event seemed to help some students as they felt they had to cope and provide an appropriate level of care – a key skill for practice.

*“I learnt on the spot and I’m not particularly good at pads and when I did it, I did it right”*

*G2 L81-82 S4 LM*

Despite the reported difficulties posed by the environment, many were keen to explain how much they felt they had learned from other clinicians. Some took the opportunity to observe new techniques and approaches both to patient assessment and provision of care from other professionals.

*“this man came in and had fallen over and it looked like his finger was broken so you’d then go and just talk to one of the doctors, and then you learn from it as you listen to how they assessed it”*

G4 L234-6 S3 LM

While not yet qualified, many students reported feeling fully engaged with the reciprocal nature of the interprofessional learning process, where other professionals were also able to learn from them. Thus, the potential for learning appeared to be reciprocal for many of those volunteering:

*“they* {referring to other professions} *learned a lot off of us... They didn’t know what we could do”*

G1 L314 S2 LM

*“We sort of bounced off of each other and learned from each other”*

G3 L313 S2 LM

While the students’ feedback was broadly positive, some moderating effects adversely affecting learning were also reported. For example, some students found working with an unfamiliar team in a different clinical environment prevented them from learning as much from this volunteering opportunity as they would have wished. The environment was difficult as space was limited, particularly when lots of runners were in situ with different clinicians trying to work together. The environment, psychosocial burden, physical demands and time pressures, coupled with students’ own expectations, meant for some that the event was difficult to cope with. In turn, this limited their ability to learn. Some students reported a need to find a role they were comfortable with and gain confidence as the day progressed, highlighting how individual learners cope and adapt in different ways:

*“I felt overwhelmed by the chaos… so I was based outside and I had an absolutely fabulous day… it was positive for me to feel as if I was making a difference”*

*G1 L142-45 S5 LM*

## Discussion

To the best of our knowledge, this is the first qualitative exploration of podiatry students’ experience of volunteering in an interprofessional context at a mass participation major sporting event. The five emergent themes highlight that the experience was generally conducive to learning new skills and implementing current knowledge. However, aside from the enjoyment and sense of accomplishment gained, a series of challenges were perceived.

Pre-registration student learning is usually formalized within a modular structure rather than an informal interprofessional setting [47]. Voluntary activities such as this appear to be one way of filling often unknown gaps in learning that can be identified by students. Our work highlights the value of interprofessional (and often diverse) placement settings [27, 48]. and underlines the value of co-produced learning needs identified by clinical staff and learners working together. As reported by previously by Holdsworth [49], we found that an individual’s confidence plays a part in how immersed students became and how they perceive and embrace the available learning opportunities. Our findings do mirror those of others, who report positive effects of volunteering on self-esteem, sense of purpose, and feelings of engagement [21–23, 49–51]. Many of the qualified podiatrists present in the wider medical team in a voluntary capacity were university alumni, suggesting that there are continued rewards for those who volunteer, as suggested in a previous model [50].

Interprofessional learning was a key theme of the students’ experience and El-Awaisi et al. [52], highlight the need for facilitation of learning by translating potential problems into learning opportunities. Examples of ‘reactive learning’ [53] were clearly reported by some students. The mass participation nature of a marathon, where the number of people seeking treatment and the nature of complaints are impossible to control [35], also appears to be a catalyst for this type of learning. Equally, this could be true of the more traditional, structured clinical setting where urgent and unexpected presentations can also occur. For example, Charcot foot (a serious complication most frequently seen in the insensate diabetic foot [54]), that may present in a musculoskeletal clinic. Consequently, the identification of expectations and matching of learners’ assumptions in a clinical learning setting, is both important and challenging [27, 55]. It may not be sufficient to simply communicate expectations in a new or diverse setting, and a process of negotiation, where learners (or volunteers in this context) can articulate their views and fears, is recommended. Unless expectations are shared and agreed, students may miss key learning opportunities; particularly in the realistic, complex experience of an interprofessional setting [27, 56, 57]. For instance, time pressures may mean staff are not always able to support students to identify and maximise their learning opportunities. This challenge is even more acute when preparing for a dynamic event or setting where the situation may change rapidly. Nevertheless, our respondents reported how useful they found the various learning opportunities, even though these opportunities were often unanticipated and difficult to prepare for.

This volunteering experience shed light on different interdisciplinary learning opportunities – problem-based, practice-based, exchange-based and observation-based – all of which were reported by our participants. As previously reported [49], this variety of learning opportunities aided our participants to solve complex issues and consequently increased their confidence as podiatry practitioners. The informal, often social, communication between professionals is reported to be key to building interprofessional working relationships [8]. The marathon environment and co-location of health professionals fostered this type of cohesion and was an important consideration for developing successful reciprocal learning, matching previous findings [47, 56, 57]. Whilst interprofessional learning is actively promoted both nationally and internationally [58], it can be difficult to embed such opportunities successfully throughout a curriculum [59]. Volunteering events such as a marathon can offer valuable interdisciplinary potential [14]. However, there are practical challenges associated with timetabling and resource management, whilst attempting to foster a sense of professional identity [60, 61].

**Fig. 2:**
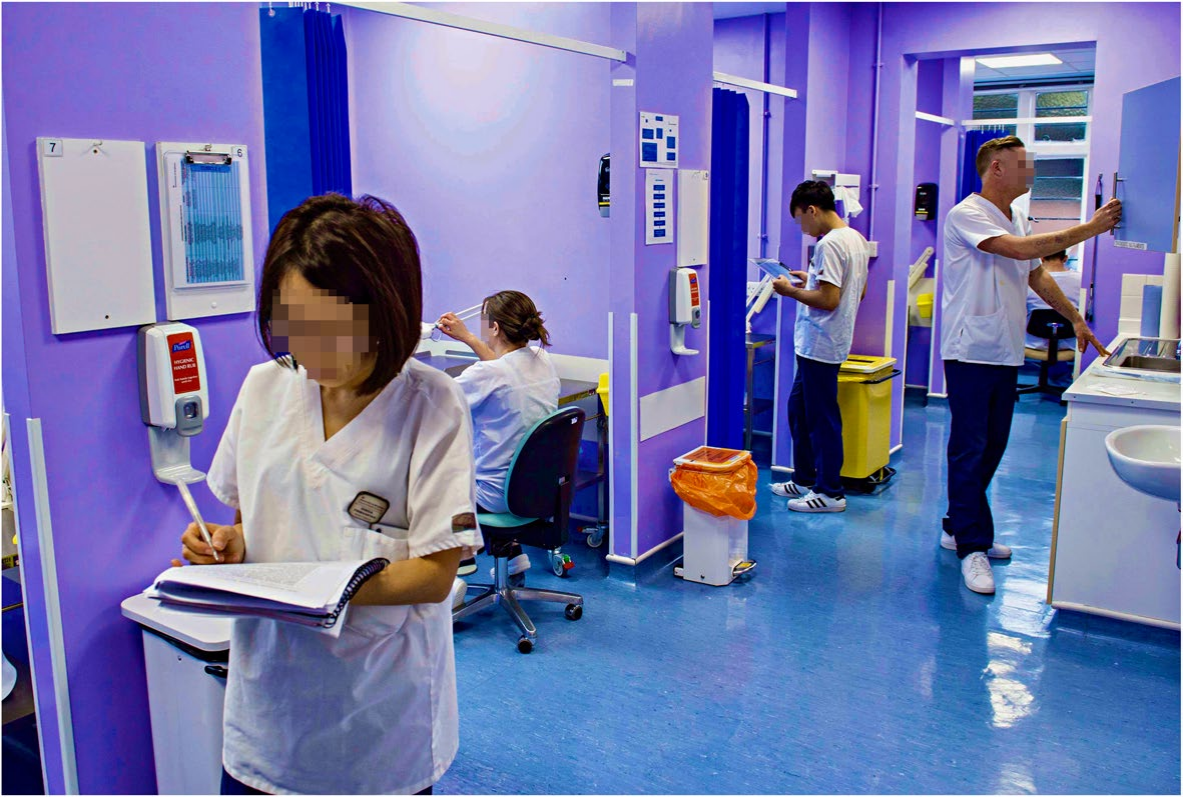
Students’ usual clinical environment. This image illustrates the University clinic in which students undertake a significant proportion of their clinical education

There is little disagreement that learning in the clinical setting is vital and consequently the environment is key to offering a series of different components to facilitate the achievement of learning outcomes and professional accreditation regulations [62, 63]. Educational establishments rightly invest considerable time and effort to audit and ensure the safety and suitability of clinical placements. Given the desire to provide a well-managed, equitable clinical educational experience, learners are not often faced with the need to adapt to continually changing pressures. However, these are the very skills clinicians require in practice. In a marathon setting, the clinical environment consists of a temporary field tent with limited supplies/facilities, in contrast with the usual more finely tuned clinical teaching environment (Fig. 2). Consequently, respondents identified how they were required to adapt their clinical practice to ensure adequate health and safety practice (such as infection control) in the ‘field’ setting. While appropriate advice was freely available for all marathon participants in the events where our students volunteered [64, 65], there remained a need for all volunteers to be able to manage runners who may have hidden medical risk factors and/or could be at risk of sudden deterioration, following the demands of a completed marathon. Understandably, our respondents initially found switching between their usual clinical environment and a different setting, quite challenging. Equally, our findings revealed that many respondents were willing to ‘test’ themselves in an unfamiliar setting, while making a positive community contribution. It would be valuable to identify whether this experience was subsequently advantageous to graduates in practice who are required to adapt to different unfamiliar surroundings (e.g., ward visits or domiciliary practice).

In terms of strengths and limitations we recognise that three members of the research team (SO, DW, PR) are podiatrists and employees of the participating university. We acknowledge that this may have influenced our interpretation of the results and led us to place greater emphasis on certain factors. The robust methodology employed [66] coupled with respondent validation [42], provides assurance of the trustworthiness of data interpretation. The inclusion of more than one cohort of students at two different marathon events gives further confidence that we are not simply reporting a ‘one off’ finding. Nevertheless, we recognise that, by their nature, mass participation sporting events may vary due to factors that cannot be controlled. For example, the clinical complaints that present on a hot, sunny day, such as heatstroke, will vary from those at a cold, wet event, where hypothermia can be a real risk [38]. Consequently, student experience can vary intensely between events. Whilst this may limit generalisability, the rich, thick textual descriptions offer a representative account that was not unidimensional; but identified positive and negative aspects of the experience. We recognise that this work is limited to one academic institution however, including other students from other institutions would be problematic for several reasons including, different curriculum requirements, varying preparation strategies and different locations at marathon events. These variables would present a range of external confounding factors which would limit the trustworthiness of the data. We acknowledge the often, implicit ontological assumption, that volunteering has a positive purpose in terms of self-efficacy, agency and participation [67]. Consequently, our focus group schedule sought to gather the widest range of experiences. Finally, the time and expense associated with travel to and participation in volunteering events may itself ‘self-select’ those who can ‘afford’ to take part [51].

In conclusion, despite the challenges faced, students overwhelmingly reported an abundantly positive and rewarding experience. The mix of experiences accurately reflects the frantic and often unpredictable nature of these events, which both impedes and facilitates learning. Consequently, our research demonstrates the considerable challenge of student preparation across varied clinical settings, in particular settings that are new or ‘diverse’. Students reported that following this experience felt they were better able to transfer their skills to a new setting, hone existing techniques, develop and apply new clinical skills, and learn how other professionals’ approach a presenting complaint that was unfamiliar to them. Consequently, this volunteering experience filled a previously unidentified gap in clinical education. Interdisciplinary opportunities were highly valued and actively utilised by the student volunteers to enhance their current clinical competencies and develop new skills.

## Recommendations

Based on our empirical findings are three core recommendations we sought to implement within our own practice area, which others may value.

1. *Improved management of expectations prior to clinical learning is key both for students and staff facilitating wider interdisciplinary learning*.
  For example, enable learners to share their anticipations and agree what is expected with qualified colleagues/supervisors prior to placement. Other professionals may not fully appreciate the role of a different groups of clinicians and may value the identification of areas where co-working can occur and build this into the placement experience.
2. *Encourage development and application of best practice in unfamiliar settings*.
  For example, facilitating learners to be able to apply techniques in different areas of practice to aid their future development as clinicians, where they will encounter a range of settings. This might be undertaken as a virtual placement when ‘hands-on’ opportunities are limited.
3. *Adding tasks to handbooks/virtual learning environments that challenge the traditional clinical teaching setting and that require deeper understanding and application of underpinning reasoning, not simply following a set protocol*.
  For example, challenge learners how to maintain high quality infection control without the use of conventional supplies such as a sterile wound dressing pack, requiring learners to understand the concepts of infection control and not rely on familiar equipment

## Supplementary Information

**Additional file 1.** Focus group schedule.

## Data Availability

Original transcripts are available on reasonable request to the corresponding author.
