# Supplementary material for: “I loved it, absolutely loved it” a qualitative study exploring what student podiatrists learn volunteering as part of an interprofessional medical team at a marathon
Source: J Foot Ankle Res. 2023 Feb 20;16:7. doi: 10.1186/s13047-023-00607-1 (PMC9939373; doi:10.1186/s13047-023-00607-1)
Supplement: Supplementary file 1 — Additional file 1. Focus group schedule. [file 13047_2023_607_MOESM1_ESM.docx]

**Additional file 1 – Focus group schedule**

**Can you start by reviewing your experience of your marathon event?**

Prompts:

How was the day set up?

How did it work? - walk me through your day

Did you feel integrated and part of the medical team as a whole?

**What were the expectations that you had beforehand?**

Prompts:

what were you expecting to see in terms of injuries and clinical problems?

**What was your experience from an educational perspective?**

Prompts:

Do you feel like you learned something from participating in the marathon?

Did you see things from a different perspective?

Did you learn anything from it?

**Do you feel you picked up a significant amount from other professions/groups?**

Prompts:

Have there been things that you’ve taken away from them and brough back into your clinical practices?

Or has your experience been very much for that specific patient groups?

Do you feel this has influenced your clinical reasoning skills at all?

Do you feel you maybe have a different knowledge base to what you had before?

**Do you feel like you were able to teach other professions/groups?**

Prompts:

Do you feel they picked up on what you do & how did you do it?

**In terms of the learning experience was there anything that you felt impeded or interrupted your learning experience?**

Prompts:

Is there anything that you feel like, ‘well actually if someone had done something about that, then maybe I would have learned more from this experience’? “

**What was the preparation beforehand?**

Prompts:

Did you look at things you might see and how to treat them?

If there were things you could change about this experience to make it a better experience what would those be?

Would you do it again?

As fully fledged graduated podiatrists when it comes around again next year and they ask for people to do it would you again?
